# Proteomics-Metabolomics Combined Approach Identifies Peroxidasin as a Protector against Metabolic and Oxidative Stress in Prostate Cancer

**DOI:** 10.3390/ijms20123046

**Published:** 2019-06-21

**Authors:** Jodi Dougan, Ohuod Hawsawi, Liza J. Burton, Gabrielle Edwards, Kia Jones, Jin Zou, Peri Nagappan, Guangdi Wang, Qiang Zhang, Alira Danaher, Nathan Bowen, Cimona Hinton, Valerie A. Odero-Marah

**Affiliations:** 1 Center for Cancer Research and Therapeutic Development, Department of Biological Sciences, Clark Atlanta University, Atlanta, GA 30314, USA; jdougan@cau.edu (J.D.); ohuod.hawsawi@students.cau.edu (O.H.); liza.burton@students.cau.edu (L.J.B.); gabrielle.edwards1@students.cau.edu (G.E.); kia.jones@students.cau.edu (K.J.); jzou@cau.edu (J.Z.); pnagappan@cau.edu (P.N.); adanaher@cau.edu (A.D.); nbowen@cau.edu (N.B.); chinton@cau.edu (C.H.); 2 Department of Chemistry, Xavier University, New Orleans, LA 70125, USA; gwang@xula.edu (G.W.); qzhang1@xula.edu (Q.Z.)

**Keywords:** PXDN, oxidative stress, metabolome, apoptosis, prostate cancer

## Abstract

Peroxidasin (PXDN), a human homolog of *Drosophila* PXDN, belongs to the family of heme peroxidases and has been found to promote oxidative stress in cardiovascular tissue, however, its role in prostate cancer has not been previously elucidated. We hypothesized that PXDN promotes prostate cancer progression via regulation of metabolic and oxidative stress pathways. We analyzed PXDN expression in prostate tissue by immunohistochemistry and found increased PXDN expression with prostate cancer progression as compared to normal tissue or cells. PXDN knockdown followed by proteomic analysis revealed an increase in oxidative stress, mitochondrial dysfunction and gluconeogenesis pathways. Additionally, Liquid Chromatography with tandem mass spectrometry (LC-MS/MS)-based metabolomics confirmed that PXDN knockdown induced global reprogramming associated with increased oxidative stress and decreased nucleotide biosynthesis. We further demonstrated that PXDN knockdown led to an increase in reactive oxygen species (ROS) associated with decreased cell viability and increased apoptosis. Finally, PXDN knockdown decreased colony formation on soft agar. Overall, the data suggest that PXDN promotes progression of prostate cancer by regulating the metabolome, more specifically, by inhibiting oxidative stress leading to decreased apoptosis. Therefore, PXDN may be a biomarker associated with prostate cancer and a potential therapeutic target.

## 1. Introduction

According to Global Cancer Incidence, Mortality and Prevalence (GLOBOCAN) 2018 database, 18.1 million new cancer cases and 9.6 million cancer deaths were estimated for 38 cancers worldwide, with an average risk of getting cancer before the age of 75 at 20%, in addition to a 10% risk of dying from cancer [1]. Moreover, in 2018. prostate cancer was the most frequently diagnosed cancer in men in 12 regions of the world followed by lung cancer, however, death from prostate cancer was second to lung cancer [1]. Prostate cancer is the second leading cause of cancer-related deaths among males in the United States [2]. Most men who die of prostate cancer present with hormone refractory and bone metastatic disease, and currently one treatment option for bone metastatic prostate cancer is bisphosphonate drugs such as Zolendronic acid [3]. However, these treatments have resulted in adverse side effects such as osteonecrosis of the jaw (ONJ) [4]. Patients with early-stage prostate cancer are usually treated with androgen deprivation therapy (ADT), however, resistance to ADT eventually develops [5]. Recently, the new-generation androgen pathway targeting drugs, that includes nonsteroidal antiandrogen (enzalutamide) and CYP17A1 inhibitor (abiraterone), have been approved by the U.S. Food and Drug Administration (FDA) for the treatment of hormone refractory prostate cancer; unfortunately, development of resistance to these new treatments has also been observed [5]. Mechanisms of resistance in prostate cancer include androgen receptor (AR) mutations, AR amplification, AR splice variants, AR bypass pathways, drug efflux alterations and inhibition of cell death [6]. More studies are needed to understand the biomarkers associated with aggressive prostate cancer that may reveal novel targets. 

Peroxidasin (PXDN) is the human homolog of *Drosophila* PXDN; in *Drosophila* it has be shown to play a role in embryonic development [7]. PXDN belongs to a family of heme-containing peroxidases that catalyze oxidation of various substrates, mainly utilizing the reactive oxygen species (ROS), hydrogen peroxide (H_2_O_2_), in the formation of hypohalous acids [8]. In cardiovascular tissue PXDN scavenges H_2_O_2_, a product of NADPH oxidase (Nox) activity, and uses it in the formation of hypochlorous acid (HOCl), which subsequently leads to endothelial cell death [8]. It has been shown that peroxidase enzymes are involved in cell adhesion and the formation of extracellular matrix (ECM) and that the oxidant agents secreted by the peroxidases can damage ECM [9]. A link between ECM proteins and PXDN revealed that PXDN acts through catalyzing sulfilimine bonds in collagen IV, an integral component of the basement membrane. 

PXDN is overexpressed in several cancers including ovarian, bladder and esophageal cancer [10,11,12] and has been associated with metastatic melanoma and brain tumors [13,14]. However, its expression has not been reported in prostate cancer. We have previously discovered an upregulation in PXDN mRNA when Snail transcription factor was overexpressed in prostate cancer cells [15]. 

Tumor cells switch from oxidative phosphorylation to glycolysis (Warburg effect) which promotes chemoresistance, therefore, metabolic reprogramming has become a major area of cancer research. The glycolytic pathway produces intermediates that supply building blocks for biosynthesis of macromolecules, such as the reduced form of nicotinamide adenine dinucleotide phosphate (NADPH) and ribose sugars for nucleotides, while excessive lactic acids from glycolysis promote tumor invasion [16,17].

In this study, we found that PXDN expression increases with prostate cancer progression, and that knockdown of PXDN leads to increased apoptosis possibly via upregulation of H_2_O_2_ in prostate cancer cells. We also present proteomics and metabolomics data indicating that PXDN knockdown activates a number of pathways associated with oxidative stress, including the nuclear factor erythroid 2-related factor 2 (Nrf2/NFE2L2) oxidative stress signaling pathway, and decreased nucleotide biosynthesis. We propose that PXDN plays a role in prostate cancer progression and may be a potential biomarker that will be useful for therapeutic targeting of prostate cancer. 

## 2. Results

### 2.1. PXDN Expression Increases with Prostate Cancer Progression

**Figure 1:**
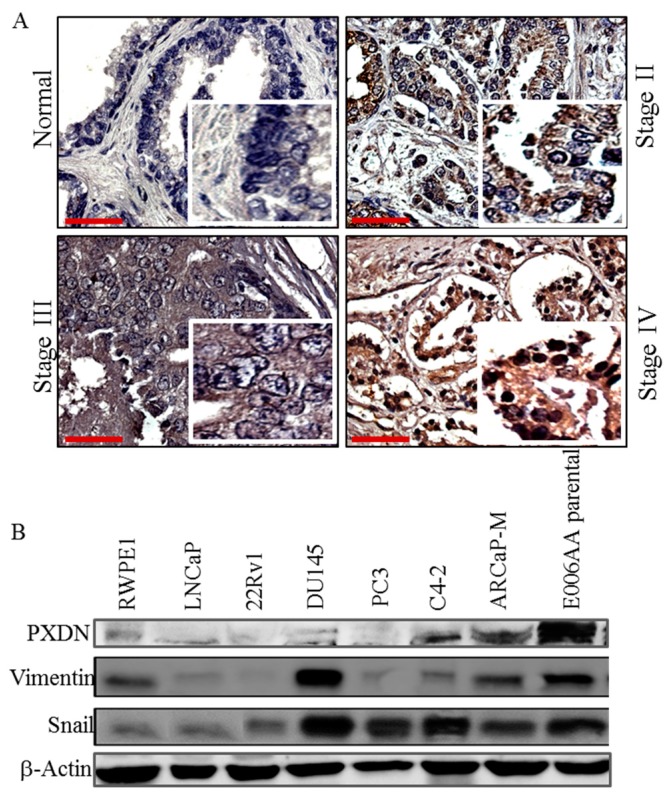
PXDN expression increases with prostate cancer progression. (**A**) Immunohistochemical (IHC) analysis was performed using a 96-core prostate adenocarcinoma tissue microarray. Representative images of PXDN in various stages of prostate cancer show that PXDN increases with tumor progression. Bar represents 50 µM. (**B**) Western blot analysis was performed on RWPE1 normal transformed epithelial cell line and various prostate cancer cell lines with antibody against PXDN or mesenchymal markers Snail and vimentin. Actin was utilized as a loading control. Data are representative of at least 3 independent experiments.

We utilized a prostate tissue microarray to analyze the expression of PXDN by immunohistochemistry. Patient information is shown in Table S1. This revealed that the expression of PXDN was not detectable in normal tissue while it increased with the stage of prostate cancer (Figure 1A). Moreover, western blot analysis for PXDN in a panel of prostate cell lines showed that PXDN expression was high in certain cells expressing mesenchymal markers Snail and vimentin, including C4-2 and ARCaP-M (mesenchymal), which are bone metastatic cell lines, as well as in the African-American cell line (E006AA), as compared to a human papillomavirus 18 (HPV 18) immortalized, non-tumorigenic cell line (RWPE1) cells established from normal prostate epithelial cells (Figure 1B). We noted that DU145 (a brain metastatic cell line) expressed high levels of Snail and vimentin, but low PXDN, an indication that not all cells expressing mesenchymal markers express PXDN. Therefore, PXDN expression may increase with prostate cancer progression.

### 2.2. PXDN Promotes Cell Viability and Tumorigenicity on Soft Agar

**Figure 2:**
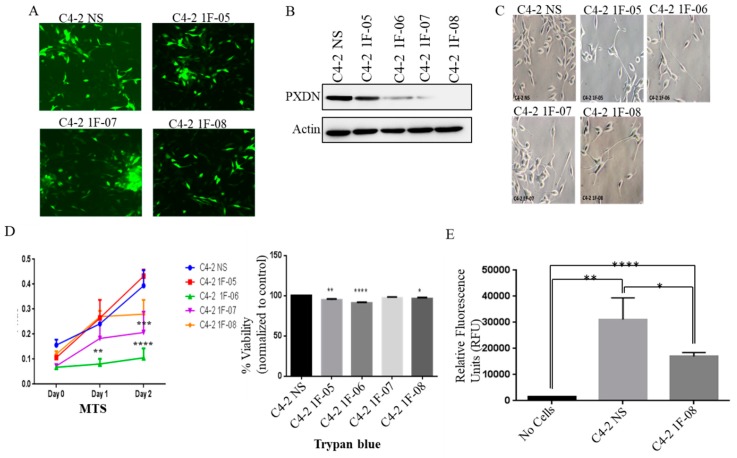
PXDN stable knockdown in C4-2 cells decreases cell viability and growth on soft agar. (**A**) PXDN was stably knocked down in C4-2 prostate cancer cells using 4 different shRNA constructs (1F-05, 1F-06, 1F-07, 1F-08) or non-silencing (NS) construct as a control. (**A**) Fluorescence microscopy (magnification at 20×) was used to detect GFP to confirm successful transduction. (**B**) Western blot analysis was performed to confirm successful knockdown of PXDN. Actin was utilized as a loading control. (**C**) Phase contrast microscopy (magnification at 20×) shows the morphology of PXDN knockdown cells. (**D**) Cell viability was investigated using MTS or Trypan blue assay. (**E**) Soft agar colony formation was measured in C4-2 NS (control) and representative C4-2 1F-08 (PXDN knockdown) cells. Values were normalized to C4-2 NS and the mean± SEM of data were obtained from three independent replicate experiments. Statistical analysis was done with GraphPad Prism; (**** *p* < 0.0001, *** *p* < 0.001, ** *p* < 0.01, * *p* < 0.05).

To study the role of PXDN, we performed a stable knockdown of PXDN in C4-2 cells, using four different short hairpin RNA (shRNA) constructs or non-silencing (NS) shRNA control. We confirmed that the transduction was successful using fluorescence microscopy to examine Green Fluorescent Protein (GFP) expression (Figure 2A). Western blot analysis revealed that the 1F-08 construct produced the greatest decrease in PXDN expression (Figure 2B), associated with a change in cell morphology, where cells exhibited long neurite-like projections (Figure 2C). To investigate the role of PXDN in cell proliferation, we performed a cell viability assay using 3-(4,5-dimethylthiazol-2-yl)-5-(3-carboxymethoxyphenyl)-2-(4-sulfophenyl)-2H-tetrazolium) (MTS) and Trypan blue assay. We observed decreased cell viability upon PXDN knockdown (Figure 2D). Interestingly, although 1F-08 cells exhibited the largest PXDN knockdown, they did not display the largest decrease in cell proliferation, however, it was significantly lower as compared to control cells (Figure 2D). We subsequently performed a soft agar colony formation assay to determine whether PXDN can affect tumorigenicity. We found that colony formation was significantly inhibited upon PXDN knockdown (C4-2 1F-08 cells) as compared to C4-2 NS control (Figure 2E). This implies overall that PXDN promotes cell viability and tumorigenicity.

### 2.3. PXDN Scavenges H_2_O_2_ in Prostate Cancer Cells and Inhibits Apoptosis

**Figure 3:**
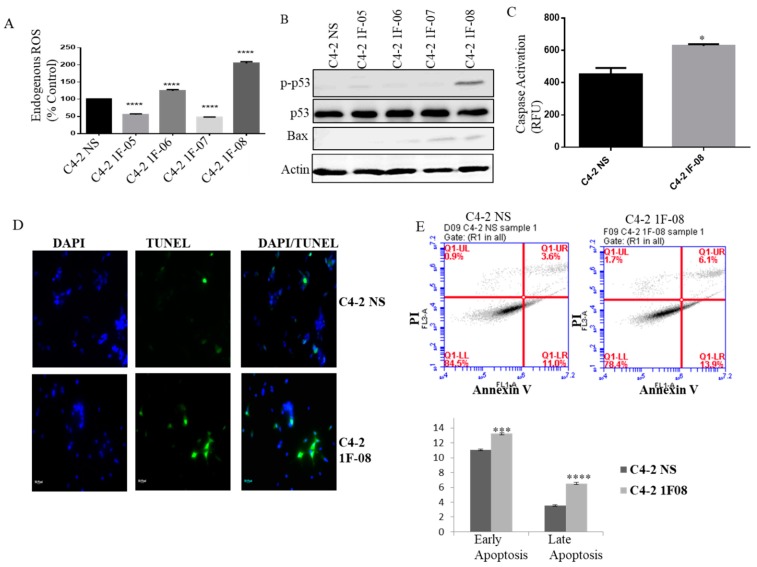
PXDN scavenges ROS and inhibits apoptosis. (**A**) ROS assay was performed to detect H_2_O_2_ in PXDN knockdown cells using ROS detection reagent CM-DCFDA for 1 h at 37 °C. 1 × 10^4^ cells per well were plated in a black walled 96-well plate and read in a spectrophotometer with excitation at 485nm and emission at 535 nm. (**B**) Western blot analysis was performed on PXDN cells to probe for pro-apoptosis related proteins p53 and Bax. Actin was utilized as a loading control. (**C**) MitoCasp assay was performed to look at caspase-3/7 activity. (**D**) TUNEL assay (magnification at 20×) was performed to detect apoptosis using the In Situ Cell Death Detection Kit. (**E**) The cells were stained with Annexin V- Alexa Fluor 488 and PI and analyzed by Flow cytometry. Values were normalized to C4-2 NS and the mean ± SEM of data were obtained from three independent replicate experiments. Statistical analysis was done with GraphPad Prism; (* *p* < 0.5, *** *p* < 0.001, **** *p* < 0.0001).

It has been shown that PXDN catalyzes the oxidation of halides by H_2_O_2_ [18]. We wanted to analyze what role PXDN plays in regulating Reactive Oxygen Species (ROS) in prostate cancer. ROS levels significantly increased when PXDN expression was silenced (Figure 3A). Therefore, PXDN in prostate cancer cells may scavenge H_2_O_2_ to possibly prevent excessively high levels of ROS that may become deleterious for prostate cancer cells. Additionally, we observed increased phospho-p53 (p-p53) and Bax expression by western blot analysis, upon PXDN knockdown, particularly in C4-2 1F-08, which has the lowest levels of PXDN (Figure 3B). MitoCasp assay was performed, which measures caspase-3/7 activity; these are effector caspases that play a role in the intrinsic apoptotic pathway. This revealed that there was significantly higher caspase-3/7 activity in C4-2 cells with PXDN knockdown (1F-08) as compared to C4-2 NS cells (*p* = 0.026) (Figure 3C). Terminal deoxynucleotidyl transferase dUTP nick end labeling (TUNEL) assay showed that there was more fluorescence in the cells containing PXDN knockdown (C4-2 1F-08) as compared to C4-2 NS control, indicating more apoptosis in these cells (Figure 3D). This was confirmed by quantitative Annexin V staining that revealed an increase in early and late apoptosis upon PXDN knockdown (Figure 3E). These results show that PXDN plays a role in scavenging ROS and also inhibiting apoptosis.

### 2.4. PXDN Regulates Oxidative Stress Pathways and Decreases Mitochondrial Membrane Potential

**Figure 4:**
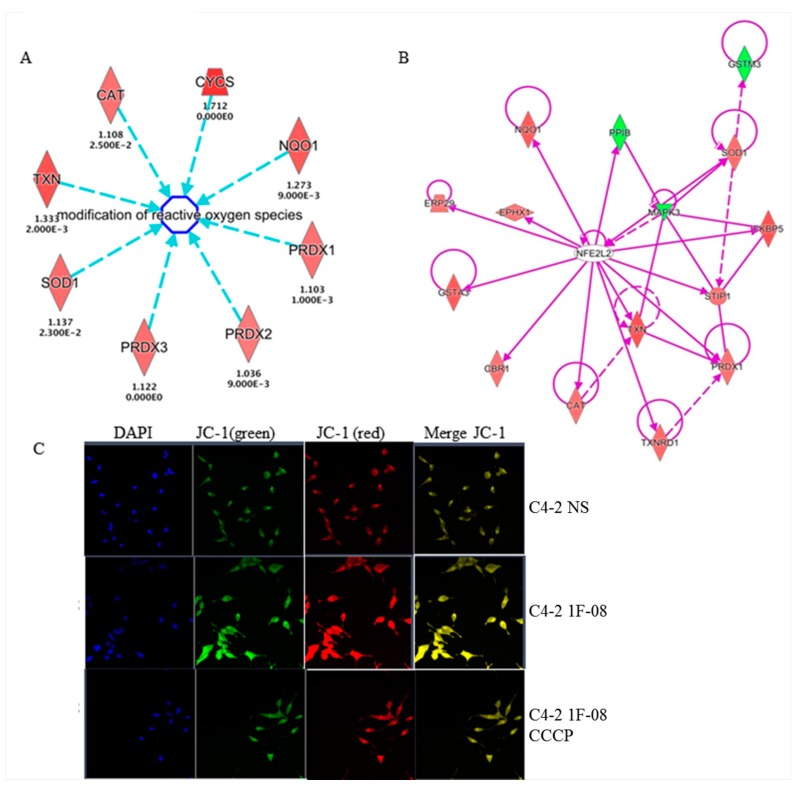
PXDN regulates oxidative stress pathways and decreases mitochondrial membrane potential. C4-2 NS or C4-2 1F-08 cells were subjected to proteomic analysis followed by ingenuity pathway analysis (IPA). (**A**) Several enzymes involved in modification of ROS were identified. Red denotes upregulated proteins following PXDN knockdown. Arrow tip towards ROS indicates upstream and regulating ROS. Blue dotted lines indicate an indirect relationship. (**B**) One key pathway predicted to be regulated was the Nrf2/NFE2L2 pathway. Purple solid lines indicate direct relationship, while dotted lines indicate an indirect relationship. Lines without arrows indicate binding. (**C**) Cells were stained with JC-1 dye to examine mitochondrial membrane potential; CCCP1 was utilized as a mitochondrial uncoupler control. Images were taken at 20×. JC-1 exists either as a green-fluorescent monomer at depolarized membrane potentials or as a red-fluorescent J-aggregate at hyperpolarized membrane potentials. Data are representative of at least 3 independent experiments.

Proteomic profiling was performed to investigate global pathways that may be regulated by PXDN in prostate cancer. C4-2 NS control or C4-2 1F-08 cells with stable PXDN knockdown were subjected to non-gel isobaric labeling Tandem Mass Tag (TMT) quantitative proteomic approach for further validation and identification of novel proteins. Differential expression (DE) analysis (*p* < 0.5) revealed that 304 proteins out of the 1194 total detected were found at significantly different levels by mass spectrometry analysis; 144 were significantly more abundant in the PXDN knockdown cell line relative to the NS control and 160 were significantly less abundant. The top UP and DOWN regulated proteins are listed in Table 1. The full list can be found in Appendix A. From the list of 304 DE proteins, the most significantly represented canonical pathways and the genes included in those pathways by Ingenuity Pathway Analysis (IPA) are listed in Table 2. Some of the pathways significantly affected were the Nuclear factor erythroid 2-related factor 2 (Nrf2), also known as nuclear factor erythroid-derived 2-like 2 (NFE2L2)-mediated oxidative stress response, phagosome maturation (possibly related to autophagy), eukaryotic Initiation Factor 2 (eIF2) signaling, mitochondrial dysfunction and gluconeogenesis I. These pathways indicate some sort of cellular stress when PXDN is knocked down. Curiously, the most significantly upregulated genes included those involved in the modification of reactive oxygen species such as catalase (CAT), superoxide dismutase (SOD1) and peroxiredoxin (PRDX1, 2, 3) (Figure 4A). This suggests that these proteins may have been triggered to attempt to scavenge the ROS released when PXDN was silenced. More support comes from the fact that the most significantly enriched canonical pathway (*p* = 2.49 × 10^−8^) included a set of 15 proteins that formed a network around the NFE2L2 (also known as Nrf2) molecule involved in the oxidative stress response (Figure 4B). Additionally, staining with JC-1 dye revealed that membrane potential was increased (more red staining) in C4-2 1F-08 cells with PXDN knockdown compared to C4-2 NS control (Figure 4C), suggesting that PXDN may contribute to lowering mitochondrial membrane potential. Therefore, PXDN may play a role in preventing oxidative stress.

### 2.5. Metabolomic Analysis Indicates that PXDN Decreases Oxidative Stress

**Figure 5:**
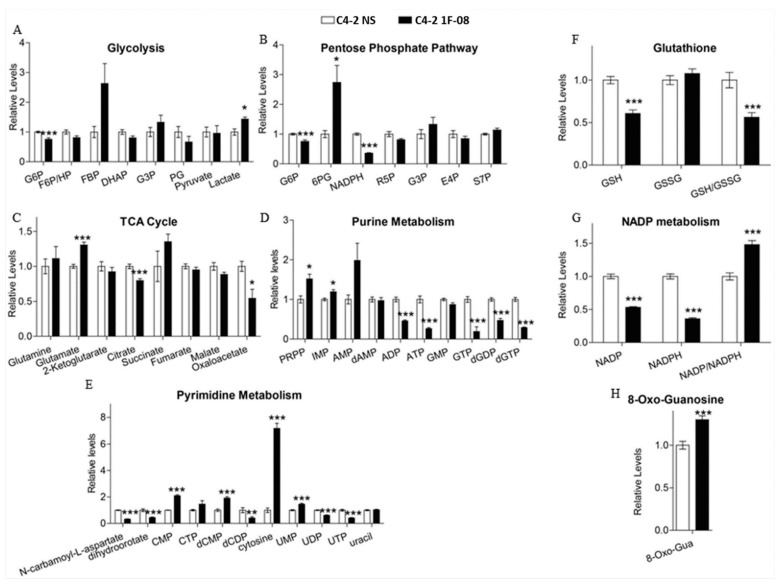
PXDN facilitates metabolic alterations. Metabolomics was performed on C4-2 NS (control) and C4-2 1F-08 (PXDN knockdown) cells. Metabolites examined included those involved in (**A**) glycolysis, (**B**) pentose phosphate pathway (PPP), (**C**) TCA cycle, (**D**) purine metabolism, (**E**) pyrimidine metabolism, (**F**–**H**) oxidative stress (GSH which is reduced glutathione; GSSG which is oxidized glutathione; 8-oxoguanosine; NADP:NADPH). Values were obtained from three independent replicate experiments and the mean± SEM of data were plotted graphically compared to C4-2 NS. Statistical analysis was done using GraphPad Prism; (*** *p* < 0.001, ** *p* < 0.01, * *p* < 0.05).

We further investigated the effect of PXDN knockdown on global metabolic changes by testing the polar metabolite profiles of cells using LC-MS/MS metabolomics. Metabolites were extracted from C4-2 NS or 1F-08 (PXDN knockdown) cells, followed by sample processing, data acquisition, and analysis. Analysis of individual metabolites showed that PXDN knockdown led to significant alterations in the metabolites from committed steps in glycolysis and the pentose phosphate pathway (PPP), including glucose-6-phosphate (G6P) and 6-phosphogluconate (6PG), as well as low reduced form of nicotinamide adenine dinucleotide phosphate (NADPH) (Figure 5A,B). These changes indicate glycolytic alterations and reduced metabolite flux downstream of 6PG in PXDN knockdown cells. The tricarboxylic acid (TCA) cycle metabolites were only moderately altered upon PXDN knockdown (Figure 5C). The oxidation of 6PG is the first committed step of PPP that is required for NADPH production and the biosynthesis of ribonucleotides, the building blocks for nucleic acid synthesis [24]. We observed low NADPH, high phosphoribosyl pyrophosphate (PRPP) and inosine monophosphate (IMP) (Figure 5D); during purine biosynthesis, PRPP is converted into IMP. There was also relatively low di- and tri-phosphates of purine nucleotides (Figure 5D). This all indicates that PXDN knockdown increases oxidative stress and inhibits purine biosynthesis. Cytosine was relatively high, while a decrease in uridine diphosphate (UDP) and uridine triphosphate (UTP) levels also indicated low pyrimidine biosynthesis in PXDN knockdown cells (Figure 5E). In addition to low NADPH, PXDN knockdown cells showed low Glutathione (GSH) and GSH/oxidized Glutathione (GSSG) ratio, but 8-Oxo-Guanosine (8-Oxo-Gua) levels were high (Figure 5F–H). Though NADP and NADPH levels decreased, there was an increase in the NADP/NADPH ratio (Figure 5G), suggesting that PXDN loss may enhance oxidative stress which corroborates proteomics data. Therefore, PXDN may be required for prevention of oxidative stress, and promotion of nucleotide biosynthesis.

## 3. Discussion

There are few reports on PXDN in cancer and no current reports on the function of PXDN in prostate cancer. Therefore, we sought to investigate the role PXDN may play in prostate cancer. We found increased expression of PXDN in prostate cancer tissue concurrent with tumor stage of prostate cancer patient tissue, while expression was undetectable/low in normal prostate epithelial tissue. PXDN has also been shown to be increased in melanoma as well as brain, kidney, and breast cancers [25,26]. PXDN catalyzes H_2_O_2_-driven oxidation and therefore usurps H_2_O_2_ [7], yet ROS is generally higher in cancer [27,28]. We speculated that increased PXDN in prostate cancer may still scavenge ROS to prevent deleterious overproduction. We confirmed this speculation by showing that stable PXDN knockdown in C4-2 prostate cancer cells led to increased ROS levels, suggesting that PXDN is still acting as a ROS scavenger. Interestingly, proteomic analysis revealed an increase in a number of pathways that suggest increased oxidative stress following PXDN knockdown including: (1) Nrf2/NFE2L2-mediated oxidative stress response following PXDN knockdown. Nrf2 is a basic-region leucine zipper transcription factor that binds to the antioxidant-response element (ARE) to mediate expression of key protective enzymes, and it is reported to be decreased in human prostate cancer [29]. (2) The eIF2 signaling pathway, which has also been implicated in protection against ROS [30]. (3) Mitochondrial dysfunction, which may promote cell death. It has been shown that ROS can initiate the apoptotic cascade via increasing the permeability of the mitochondrial membrane [21]. (4) Phagosome maturation, which involves the fusion between phagosomes and intracellular organelles and has been implicated in the elimination of cells that have undergone apoptosis [31]. (5) Gluconeogenesis, which is the biosynthesis of glucose and the reverse of glycolysis. 

The most upregulated protein following PXDN knockdown was Down syndrome cell adhesion molecule (DSCAM), which is expressed in the developing nervous system with the highest level in fetal brain [19]. DSCAM has been implicated in neurite patterning, including axon branching [32,33]. Since we observed long neurite-like processes in C4-2 cells when PXDN was knocked down in this study, it is interesting to speculate whether this may be due in part to DSCAM upregulation. This may imply that PXDN inhibits neurite extensions which needs to be further explored, whether this may play a role in processes such as neuroendocrine differentiation of cancer cells. Another protein that was significantly upregulated following PXDN knockdown was protein tyrosine phosphatase type IVA 2/ phosphatase of regenerating liver (PTP4A2/PRL-2); this is a prenylated protein tyrosine phosphatase with oncogenic activity that has been proposed to drive tumor metastasis [20]. This may imply that PXDN may play a role as a metastasis suppressor, although in our studies it increased tumorigenicity in a colony formation assay. Peroxiredoxins (PRDXs) are a fascinating group of thiol-dependent peroxidases that are responsible for functions such as protecting cells against oxidative DNA damage and genomic instability, regulating cell signaling associated with H_2_O_2_, and influencing cell differentiation and proliferation, immune responses and apoptosis [22]. PRDX1, PRDX2, PRDX3, PRDX5 and PRDX6 are localized in the cytosol, in the mitochondria, in the nuclei and in the peroxisomes, whereas PRDX4 is mainly present in the endoplasmic reticulum or it is secreted [22]. Our proteomic data indicated that PRDX4 is upregulated upon PXDN knockdown, which would correlate with the increased H_2_O_2_ observed in these cells. Additionally, in other types of epithelial cancers such as oral cavity squamous cell carcinoma, breast cancer, ovarian cancer, and lung cancer, overexpression of PRDX4 correlates with the metastatic potential [22] again implicating an alternative possible role for PXDN in regulating metastasis. Another protein also associated with metastasis is mitochondrial solute carrier family 25 (SLC25), which was also upregulated when PXDN was silenced. Knockdown of SLC25 in colon cancer cells suppressed proliferation, migration, and invasion in vitro and reduced the formation and metastasis of tumors in vivo [23], suggesting it is a metastasis promoter that can be suppressed by PXDN. However, PXDN has been associated with metastatic melanoma and brain tumors [13,14]. Therefore, the intriguing role PXDN may play in prostate cancer needs to be explored further. It would be interesting to further dissect the role of PXDN in tumorigenesis as compared to metastasis.

Another upregulated protein following PXDN knock-down, Aldehyde dehydrogenase 1 (ALDH1) is a marker of stem cells and cancer stem cells, and it acts as a detoxifying enzyme that oxidizes aldehyde into carboxylic acid and converts retinol into retinoic acid [34]. It is ubiquitously distributed in various tumors, including breast cancer, non-small-cell lung cancer, laryngeal cancer, ovarian cancer, and gastric cancer [34]. Many studies have shown that ALDH1 is a marker of breast cancer stem cells [34]. It is possible that it is upregulated as a response to high ROS induced by PXDN knockdown. This is supported further by our proteomics data showing that Nrf2 protein was also increased following PXDN stable knockdown. Nrf2 is a transcription factor that responds to environmental hazardous insults, including reactive oxygen species (ROS) [35]. 

Gluconeogenesis significantly suppresses aerobic glycolysis and influences other metabolic pathways in cancer cells, including the TCA cycle, oxidative phosphorylation, the pentose phosphate pathway, glutaminolysis, and serine and nucleotide biosynthesis [36]. Some types of cancers hijack gluconeogenesis, which promotes metabolic flexibility by allowing the utilization of non-carbohydrate sources for biosynthesis and redistributing glucose flux for antioxidant production [36]. Our metabolomics studies supported the proteomics studies in showing that PXDN influences the gluoconeogenesis pathway. Metabolomics performed in this study did reveal a moderate change in some of the metabolites in the TCA pathway, but the data was stronger in support of PXDN regulating metabolites that prevent oxidative stress and promote nucleotide biosynthesis, which would be essential for increased DNA replication in highly proliferating cells. Indeed, our results showed that PXDN knockdown led to a slight but significant decrease of cancer cell proliferation, suggesting that PXDN promotes cell proliferation; this is similar to a study showing that PXDN promotes proliferation of vascular smooth muscle cells as well as lens epithelium during eye development [37,38]. PXDN knockdown also led to decreased tumorigenesis as shown by soft colony formation assay; this suggests that PXDN can promote tumorigenicity. 

With PXDN knockdown, oxidative stress may lead to apoptosis and our results revealed an increase in B-cell lymphoma 2 Associated X, Apoptosis Regulator (Bax) and phosho-p53, both known pro-apoptotic markers, following PXDN knockdown. p53 directly activates Bax [39] while Bax has been shown to play a direct role in mitochondrial membrane permeabilization [40]. By utilizing JC-1 staining, we did observe that PXDN knockdown also increased mitochondrial potential indicative of mitochondrial damage. Moreover, Mitocasp assay revealed increased caspase-3/7 activation while a TUNEL assay indicated increased apoptosis following PXDN knockdown. Therefore, PXDN may prevent mitochondrial damage and inhibit oxidative stress and apoptosis.

Collectively we conclude that PXDN promotes prostate cancer progression by scavenging ROS to possibly inhibit oxidative stress and apoptosis. Therefore, increased PXDN expression may be a potential biomarker for aggressive prostate cancer, and the inhibition of PXDN may be a possible therapeutic target for aggressive prostate cancer. 

## 4. Materials and Methods

### 4.1. Cell Culture

RWPE1, DU145, PC3, 22Rv1 and LNCaP cells were from American Type Culture Collection (Manassas, VA, USA). C4-2 cells and ARCaP-M, a mesenchymal cell line, were a kind gift from Dr. Leland Chung, Cedars-Sinai Medical Center, Los Angeles, CA, USA. All these cells were grown in RPMI-1640 (Lonza, Walkersville, MD, USA) supplemented with 10% (v/v) FBS, 50 μg/mL penicillin, and 100 μg/mL streptomycin (Mediatech, Manassas, VA, USA), except for RWPE1 cells, which were grown in Keratinocyte-SFM media supplemented with keratinocyte supplements (Thermo Fisher Scientific, Grand Island, NY, USA). The African American prostate cancer cell line E006AA parental was a kind gift from Dr. Shahriar Koochekpour of Roswell Park Cancer Institute (Buffalo, NY, USA). These cells were grown in DMEM (Mediatech, Herndon, VA, USA) with 10% (v/v) fetal bovine serum (FBS), 50 μg/mL penicillin, and 100 μg/mL streptomycin. 

### 4.2. Western Blot

Western blot was performed as described previously [10]. Briefly, cell lysates (30–50 μg) were subjected to SDS-polyacrylamide gel electrophoresis (SDS PAGE) and then subsequently transferred to pure nitrocellulose membrane (Bio-Rad Laboratories, Hercules, CA, USA. Blots were visualized with a chemiluminescence ECL detection system (EMD Millipore, Rockford, IL, USA) and analyzed using a FujiFilm LAS-3000 imager (Fujifilm, Tokyo, Japan). HRP-conjugated sheep anti-mouse, donkey anti-rabbit secondary antibodies were from GE Healthcare UK Limited (Pittsburgh, PA, USA). PXDN antibody was from Abnova (Walnut, CA, USA). Rabbit polyclonal phospho-p53 and Bax antibodies were from Cell Signaling Technology (Danvers, MA, USA), while mouse monoclonal total p53 was from Santa Cruz Biotechnology, Santa Cruz, CA, USA. Mouse monoclonal β-actin antibody was from Sigma-Aldrich (St. Louis, MO, USA).

### 4.3. Stable Knockdown of PXDN

C4-2 parental cells were plated in 12-well dishes at a density of 1 × 10^5^ cells per well in complete RPM1. The next day, cells were transduced with 2 μg of shRNA (OriGene Technologies Inc., Rockville, MD, USA) using TurboFect (Thermo Fisher Scientific, Grand Island, NY, USA) following the manufacturer’s instructions. Non-silencing shRNA (NS) was used as a control along with 4 constructs targeting different sites of PXDN mRNA; 1F-05, 1F-06, 1F-07 and 1F-08. 72 h later, selection media was added containing 1 μg/mL puromycin (EMD Millipore, Danvers, MA, USA). Successful transduction and knockdown were confirmed by detection of GFP via fluorescence microscopy (Carl Zeiss, Hanover, Germany) and Western blot analysis, respectively.

### 4.4. Proteomic Profiling of C4-2 Cells with PXDN Knockdown

For both control C4-2 NS (non-silencing), and C4-2 1F-08 (PXDN stable knockdown), proteomic profiling involving LC-MS/MS analysis was performed as previously described [41]. 

### 4.5. Metabolomics

Metabolite extraction and LC-MS/MS analysis was performed as described previously [42] for C4-2 NS (non-silencing), and C4-2 1F-08 (PXDN stable knockdown). 

### 4.6. Immunohistochemistry

PXDN expression in human prostate normal or cancerous was examined by immunohistochemistry (IHC) utilizing human prostate tissue microarray (PR953, US Biomax, Rockville, MD, USA). IHC was performed using the Avidin-biotin method using 1:200 primary antibody against PXDN as previously described [43]. Images were acquired using the Carl Zeiss Axiovision Rel 4.8 (Hanover, Germany).

### 4.7. Cell Viability Assays

C4-2 NS control (2.5 × 10^4^) or each of the 4 PXDN knockdown cells were plated in a 24-well plate in complete RPMI and incubated for 72 h. Cells were harvested and resuspended in 0.4% Trypan blue solution (Sigma, St. Louis, MO, USA. Viable cells were counted using a hemocytometer (Electron Microscopy Sciences, Hatfield, PA, USA). The number of live cells was then calculated and represented graphically. MTS assay (CellTiter 96^®^ AQueous One, Promega, Madison, WI, USA) was also utilized to assess cell viability according to the manufacturer’s instructions.

### 4.8. ROS Assay

C4-2 NS or PXDN knockdown cells were cultured to approximately 80% confluency. Cells were trypsinized, counted and re-suspended in 20 µM of CM-DCFDA (Invitrogen, Eugene, OR, USA) and incubated at 37 °C for 1 h. The reaction was stopped on ice and the cells were centrifuged. 1 × 10^4^ cells resuspended in PBS were plated in a black walled 96-well plate and read immediately at 485 nm excitation and 535 nm emission. 

### 4.9. Apoptosis Assays

For TUNEL assay, 5 × 10^3^ C4-2 NS and C4-2 1F-08 cells were plated in a chamber slide and allowed to attach overnight. To detect apoptosis, the In Situ Cell Death Detection Kit (Roche Diagnostics, Indianapolis, IN, USA) was used following manufacturer’s protocol. The slides were cover-slipped with Anti-fade (Fluoro-Gel, Electron Microscopy Sciences, Hatfield, PA, USA) before imaging using Axiovision Rel 4.8. For quantitative apoptosis, Annexin V-Alexa Fluor 488 Assay was utilized as previously described [41]. 

### 4.10. Detection of Membrane Potential

The mitochondrial membrane potential was examined by staining the C4-2 NS or C4-2 1F-08 cells with JC-1 (Thermofisher, Waltham, MA, USA), as per the manufacturer’s instructions. Briefly, cells were loaded with 5 µM JC-1 dye for 20 min at 37 °C. Carbonyl cyanide m-chlorophenylhydrazone (0.5 µM CCCP; mitochondrial uncoupler) was included as a control. Cells were subsequently imaged using confocal microscopy (Zeiss, Hanover, Germany).

### 4.11. MitoCasp Assay

The MitoCasp assay (Cell Technology; CBA-130, San Diego, CA, USA) that we utilized measures caspase-3/7 activity at Excitation: 488 and Emission: 530. 1 × 10^6^ C4-2 NS and C4-2 1F-08 cells were plated overnight in RPMI growth media. The following day, the cells were trypsinized and cells incubated with caspase detection reagent for 1 h according to the manufacturer’s instructions for suspension cells. Following washes with wash buffer, 10,000 cells were plated in 96-well plates and fluorescence was measured using a microplate reader (488/530 nm filter set, BMG LABTECH, Cary, NC, USA). 

### 4.12. Soft Agar Colony Formation Assay

The CytoSelect 96-Well Cell Transformation Assay (Soft Agar Colony Formation) kit (Cell Biolabs; CBA-130, San Diego, CA, USA) was used to assess the colony formation ability as per the manufacturer’s instructions. Fluorescence was measured using a microplate reader (485/520 nm filter set, BMG LABTECH, Cary, NC, USA). 

### 4.13. Statistical Analysis

Statistical analyses and graphs were generated using GraphPad Prism 6.0 software (San Diego, CA, USA). * *p*-values < 0.05 were considered significant.

## Figures and Tables

**Table 1:** Top upregulated and downregulated proteins following PXDN knockdown identified using IPA.

Molecules	Fold Change
**DSCAM** (Down syndrome cell adhesion molecule); Plays a role in CNS development [19].	2.198
**PTP4A2** (protein tyrosine phosphatase type IVA, member 2, phosphatase of regenerating liver 2 (PRL2) [20]	1.941
**CYCS** (cytochrome c, somatic); Component of electron transport chain in mitochondria, also plays a role in apoptosis initiation [21].	1.712
**PRDX4** (peroxiredoxin 4); Reduces H202 and alkyl hydroxides to water and alcohol. Upregulated in prostate cancer [22].	1.663
**PTGES2-AS1** (prostaglandin E synthase 2- antisense RNA 1)	1.572
**SRRM1** (serine/arginine repetitive matrix 1)	1.57
**ALDH1A1** (aldehyde dehydrogenase 1 family member A1); Component of alcohol metabolism pathway.	−1.707
**HBA1/HBA2** (hemoglobin subunit alpha ½); Components of hemoglobin.	−1.684
**FECH** (ferrochelatase); Enzyme involved in heme synthesis.	−1.632
**TUBA1B** (tubulin alpha 1B); Upregulation associated with poor outcome in hepatocellular carcinoma.	−1.539
**SLC25A1** (solute carrier family 25); Regulates transport of citrate across inner membranes of mitochondria. Shown to play a role in inflammation induced by TNFα and IFNγ [23].	−1.509

**Table 2:** Top canonical pathways regulated by PXDN.

Top Canonical Pathways			
Name	*p*-Value	Overlap	Molecules from DE Proteins in PXDN KD Experiment in Pathway
**NRF2-mediated Oxidative Stress Response**	2.49 × 10^−8^	8.3% (15/180)	GSTA3,PPIB,PRDX1,GSTM3,NQO1,SOD1,TXNRD1,ERP29, STIP1,MAPK3,CAT,TXN,FKBP5,CBR1,EPHX1
**phagosome maturation**	1.27 × 10^−6^	8.7% (11/127)	NSF,CALR,TUBA1B,PRDX1,TUBB4B,RAB7A,CANX,TUBB4A, TUBB,PRDX6,PRDX2
**EIF2 Signaling**	1.71 × 10^−6^	7.0% (13/187)	RPS28,EIF1,RPLP1,RPL13,RPL3,MAPK3,RPS27L,RPLP2,RPS10, RPS3,RPS12,RPL7,EIF3K
**Mitochondrial Dysfunction**	1.82 × 10^−6^	6.9% (13/188)	SDHA,PRDX3,NDUFA5,NDUFA6,CAT,UQCRC2,CYCS,VDAC1, OGDH,NDUFS3,NDUFAB1,COX4I1,VDAC2
**Gluconeogenesis I**	2.63 × 10^−6^	15.2% (7/46)	PGK1,PGAM1,ALDOA,GAPDH,MDH1,MDH2,ALDOC

## Supplementary Materials

Supplementary materials can be found at https://www.mdpi.com/1422-0067/20/12/3046/s1.

## Abbreviations

H_2_O_2_	Hydrogen Peroxide
PXDN	Peroxidasin
ROS	Reactive Oxygen Species
ARCaP	Androgen-Repressed Human Prostate Cancer Cell Line
PRPP	Phosphoribosyl Pyrophosphate
IMP	Inosine Monophosphate
UDP	Uridine Diphosphate
UTP	Uridine Triphosphate

## Appendix A

The mass spectrometry proteomics data have been deposited in the Peptide Atlas (http://www.peptideatlas.org/PASS/PASS01244)

To access files via FTP, use credentials:

Servername: ftp.peptideatlas.org

Username: PASS01244

Password: KN5843kr
