# Supplementary material for: Proteomics-Metabolomics Combined Approach Identifies Peroxidasin as a Protector against Metabolic and Oxidative Stress in Prostate Cancer
Source: Int J Mol Sci. 2019 Jun 21;20(12):3046. doi: 10.3390/ijms20123046 (PMC6627806; doi:10.3390/ijms20123046)
Supplement: Supplementary file 1 [file ijms-20-03046-s001.pdf]

Supplemental Table S1

Table S1. Patient characteristics of prostate cancer microarray utilized in this study. 96 core prostate adenocarcinoma tissue microarray was utilized and representative patient data shown. Clinical data includes age, pathology diagnosis, stage Gleason score Type, Prostate Sera Antigen (PSA) status. T=Primary Tumor N= Regional Lymph Nodes M= Distant Metastasis.

Position	Age	Organ	Pathology diagnosis	Stage	Gleason score	TNM	Type	PSA ng/mL
G9	69	Prostate	Normal	*	*	*		*
B6	62	Prostate	Adenocarcinoma	II	7	T2cN0M1	Malignant	37.3
B10	60	Prostate	Adenocarcinoma	III	9	T3bN0M0	Malignant	40
B12	70	Prostate	Adenocarcinoma	IV	7	T4N0M0	Malignant	7
